# Reform of the Lunacy Laws

**Published:** 1886-01

**Authors:** James B Silkman


### REFORM OF THE LUNACY LAWS.

BY JAMES B SILKMAN.

Notwithstanding the vast strides now being made in every department of science, it must be admitted by every thinker, that, in medical science, which is especially one of experiment, there can be no finality ; and that, until we shall have approximated the utmost limit of that art rather than science, it is alike the duty of every philanthropist and every reformer to suggest to alienists and legislators every possible improvement in regard to the supervision and management of the insane. Nor should a free and searching criticism of the official conduct of those who have this most unfortunate class of our fellow citizens under their charge as wards of the State, be treated with stolid indifference, repelled by personal abuse, or evaded by legal artifice.

The magnitude of this subject is apparent when we realize what reliable statistics inform us, that in the various public and private asylums of this State alone there are more than 12,000 patients, sane and alleged insane, and in the United States not less—probably more than 100,000.

But this question becomes a subject of far more serious consideration, not only for every humanitarian, but every tax-payer even, in view of the astounding annual increase of the ratio of the insane over that of the increase of our entire population. So that it may be safely estimated, on the basis of such statistics as we have, that during the last fifteen or sixteen years the ratio of increase in the number of insane for our whole country has quadrupled that of the increase of the entire population. And, again, if necessary to intensify the importance of this subject, we have at hand the statement, made not long since by a distinguished alienist, one of our most popular and successful superintendents, who, before leaving this State to become the successor of the celebrated Dr. Kirkbride, of Philadelphia, told one of our highest State officers that he had seven hundred patients under him who ought to be free—seven hundred who should be earning their own support!

The lunacy laws of our 38 states and several territories may all be classified under three heads—those relating to the commitment of patients, those relating to the supervision and management of asylums, and those relating to the discharge of patients. The wrongs done and the crimes committed under the first head, though many in the aggregate, are yet relatively few, but they are in marked contrast to the great multitude of wrongs done to patients under the other two heads, in their nature and kind.

In a recent lunacy case,* Hon. Chas. F. Brown, of the Supreme Court, in his charge to the jury stated that if the plaintiff "was a perfectly sane and innocent man, who was arrested in his home and sent to the asylum, he did not think the amount of damages which he asks [$25,000] would compensate for three months confinement in an insane asylum. As far as I, myself, am concerned, I would rather go to a state prison than an insane asylum, provided I was sane. To put a sane man in company and association with lunatics is about as severe treatment as you can give him."

Hence the need of reform in the laws relating to the commitment of patients, not for the purpose of obstrucing their speedy admission in critical and exceptional cases but to prevent the recurrence of such abuses as have been too frequent of late; and this can be effected with almost a single stroke of the legislative pen. I would not advocate a jury for all cases, as they may have in Illinois and in Indiana (where they can have two juries); but whenever the patient has mind enough to demand a jury it should be granted and this privilege secured by the most stringent safeguards. With this one single provision we should seldom, if ever again, hear of a sane person kidnapped and railroaded into an asylum by interested relations.

*To be continued.*

*J B. Silkman vs. Darius G. Crosby, tried at White Plains, Sept., 1885, in which a verdict for $15,000 was rendered for plaintiff.

